# An Uncommon Presentation of Pyogenic Granuloma

**DOI:** 10.7759/cureus.12509

**Published:** 2021-01-05

**Authors:** Eder Luna-Ceron, Ana K Gómez-Gutiérrez, Cinthia Gonzalez-Hernandez, Michelle Gatica-Torres

**Affiliations:** 1 Clinical Sciences, Tecnológico de Monterrey, Mexico City, MEX; 2 Family and Community Medicine, Centro de Salud con Servicios Ampliados, Anáhuac, MEX; 3 Dermatology, Instituto Nacional de Ciencias Médicas y Nutrición Salvador Zubirán, Mexico City, MEX

**Keywords:** port-wine stain, pyogenic granuloma, lobar hemangioma, case report

## Abstract

Benign vascular neoplasms are common clinical problems encountered in the practice of primary care. Pyogenic granulomas are one of the most common benign vascular lesions in young adults. Although the physiopathological mechanism for the development of this condition is still not well understood, it has been commonly associated with several triggers such as treatment with retinoids, biological agents, invasive cutaneous therapies and trauma. The development of pyogenic granulomas on sites of vascular malformations like port wine stains has been described in the literature to occur rarely. Most of these types of cases have been studied to occur in the setting of pregnancy and after cryotherapy or pulsated laser therapy. The aim of this article is to present the case of a 21-year-old man with a recent appearance of a pyogenic granuloma within an underlying port wine stain in the posterior cervical region without any history of triggers or risk factors. Excision of the vascular lesion was done, and histopathological report confirmed the diagnosis. The objective of this manuscript is to discuss the possible mechanisms involved in the development of this uncommon presentation and to summarize the current literature related to this clinical scenario.

## Introduction

Pyogenic granuloma (PG), also known as a lobar hemangioma, is a common benign vascular neoplasm that is characterized histologically by numerous lobules of arranged capillaries and venules within fibrous and edematous stroma [1]. This lesion is usually presented in children and young adults as a red exophytic nodule or papule with an ulcerated or friable surface [1,2]. PGs are susceptible to bleeding and are typically covered by serosanguineous moisture and often appear on the surface of the scalp, cheeks, forehead, extremities, and oral mucosa [1,3]. The physiopathological mechanism involved in the development of PG is still not understood, but it has been associated with traumatic injuries, viruses, drugs, and underlying arteriovenous malformations that trigger abnormal angiogenesis by expression of growth factors and nitric acid pathway [4,5].

Port-wine stain (PWS), also known to be a subtype of nevus flammeus, is one of the most common congenital vascular malformations and appears in almost 0.3% of newborns [6]. PWS is caused as a result of a faulty vessel development during embryogenesis and is histologically characterized by the presence of an increased number of thin ectatic vessels within the papillary and reticular dermis [7]. PWS is usually developed at birth, presenting a gradual growth in size and a progressive darkening in color with persistence over time. The difference between PWS and other vascular malformations present at birth, such as salmon patches, lies in its lack of involution with age [6,7]. PWS characteristically appears as well sharply demarcated red or purple flat macules or patches that usually do not cross the midline. The surface of lesions can be uniform or acquire an increased thickness and nodularity producing a cobblestone-like texture. PWS mostly affects the head and neck but they can also involve extremities, gingiva, tongue, and extremities with less frequency [7,8].

Given the relationship of PWS with around 35 syndromes, the presence of these lesions might be related to various extracutaneous findings [6]. The most commonly portrayed related disorder is Sturge-Weber Syndrome, which is characterized by PWS following the V1 trigeminal branch distribution, ipsilateral visual defects, and leptomeningeal anomalies that result in seizures or mental impairment [7]. Pyogenic granulomas have been depicted to result as a complication of subjacent vascular distortions elicited by explicit triggers such as cutaneous treatment or pregnancy. Although spontaneous development of both lesions has been registered, this event is rarely described without the presence of a precipitating event. A case of a PG emerging from a PWS in a youngster with non-precipitating events or extracutaneous signs is described in this article.

## Case presentation

**Figure 1:**
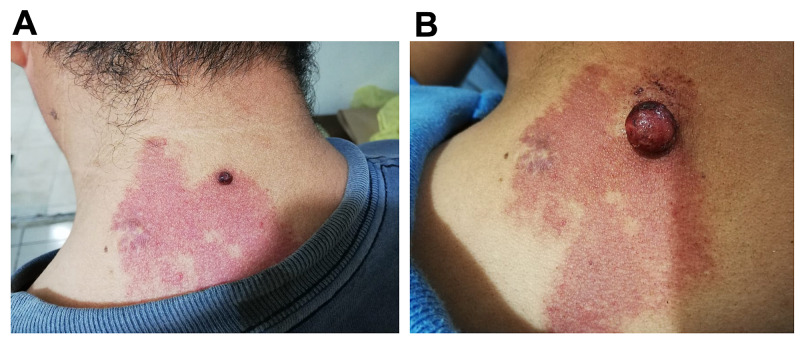
Reddish pedunculated mass with a friable surface growing over a well demarcated erythematous and micronodular patch at the posterior cervical region. (A) Lesion at the first appointment. (B) Clinical evolution of the mass two months after.

An otherwise healthy 21-year-old man was seen by his primary care clinician in 2020 with the complaint of the recent appearance of a small bleeding-pedunculated mass in the cervical posterior region from two weeks before the consultation. During the physical examination, a 0.8-cm reddish pedunculated nodule with a friable and moist surface covered by serosanguineous material was seen (Figure 1A). The mass was surrounded by a well-circumscribed erythematous patch with a slight nodular texture. The lesion did not cross the midline and was referred by the patient that this plaque has developed since birth and slightly increased in size over the years and darkened in color. The presence of this type of lesion was not found in other areas of the skin. The rest of the clinical examination including neurologic and ophthalmologic evaluation was normal.

Upon patient history, the patient denied previous treatment of the stain by any modality including pulsated laser or cryotherapy. The history of the use of retinoids, biological therapies, and trauma at the site of the lesion were also denied. The lesions were clinically compatible with a pyogenic granuloma arising over a PWS and the patient was sent to the dermatology service for excision of the mass. The patient did not have the opportunity to go for his dermatology consultation and returned to the clinic after two months of referring and increasing in size and bleeding of the lesion previously described (Figure 1B). The patient was sent for excision thereafter. Polypoid mass was excised and sent to histopathological examination that reported radially arranged numerous and dilated capillaries and stromal inflammatory infiltrate in a background of thin vessels with ectasia upon papillary dermis and the diagnosis of PG and PWS was confirmed.

## Discussion

Cases of PG arising from vascular malformations such as PWS and cherry hemangiomas have been described to occur uncommonly (Table 1). A key trigger for the co-occurrence of these lesions is the previous treatment of vascular malformations with invasive cutaneous therapies such as pulses with tunable dye lasers or cryotherapy. Several studies have described the physiopathological mechanism involved in the development of PG as a reactive process that results from the non-selective damage of both epidermal and dermal components of the skin including superficial blood vessels [9]. Laser and trauma injuries stimulate the formation of granulation tissue involving the migration of inflammatory cells and proliferation of endothelium, a process which is driven by several cytokines and mediators such as vascular endothelial growth factor (VEGF) and nitric oxide (NO) [10]. These factors have been proven to participate in the development of PG by increasing the activity of Fms-related tyrosine kinase 4 (FLT4) and NO pathway in the underlying vasculature [4]. This co-occurrence phenomenon associated with laser injury has been described to occur more commonly in the head and neck area [9,11]. Although our case presented similar topography, it did not find any evidence of previous traumatic triggers.

**Table 1:** Cases previously reported of pyogenic granulomas within underlying port wine stain lesions.

Reference	Age and Sex	Anatomical Region	Underlying Triggers	Treatment and Other Notes
Lanigan and Cotterill [9]	21-year-old female	Neck (Right side)	Continuous-wave tunable dye laser and pregnancy	Curettage and cautery
Lanigan and Cotterill [9]	17-year-old male	Forehead (Glabella)	Continuous-wave tunable dye laser + Carbon dioxide laser	Curettage and cautery
Sheehan and Lesher [11]	35-year-old male	Upper dorsum	No underlying trigger	Saucerization
Madi et al. [12]	33-year-old female	Gingival mucosa	No underlying trigger	Surgical excision
Katta et al. [13]	16-year-old female	Nose (dorsal area)	Pregnancy (24 weeks of gestation)	Shave excision
Barzegar et al. [14]	26-year-old female	Gingival mucosa	Pregnancy (24 weeks of gestation)	Surgical excision
Rodins et al. [15]	23-year-old female	Cheek (left)	Pregnancy (28 weeks of gestation)	Punch biopsy
Fernandez-Vazquez et al. [16]	52-year-old male	Neck (middle line)	Venous malformation	Surgical excision (Multiple PG, multiple recurrences)
Askar et al. [17]	25-year-old male	Posterior cervical area	No underlying trigger	Surgical excision
Swerlick and Cooper [18]	22-year-old female	Left thigh	No underlying trigger	Surgical excision
Swerlick and Cooper [18]	8-year-old male	Left shoulder	Cryotherapy injury	Cryotherapy (5 recurrences)

An important proportion of PG arising from PWS has been associated with pregnancy. Since there is a clear relationship between these entities, several authors have suggested that the increasing levels of circulant hormones such as progesterone and estrogens in pregnancy can exert the process of angiogenesis, decrease apoptosis in endothelial cells, and increased blood flow through the enlarged and ectatic vessels of PWS facilitating the delivery of pro-angiogenic factors and contributing for the development of PG [10,12]. A characteristic affection of gingival mucosa is consistent in most of the cases of co-occurrence during pregnancy [13-15]. Also, cases of PG have been shown to recur even in different anatomical locations with subsequent pregnancies [15]. The hormonal influence in the development of PG has clearly been stated and PG has been described to occur not rarely in the context of oral contraceptive consumption [16].

Our case presented unique features since it did not follow the characteristic topography and a clear trigger for the development of a PG as described previously. A small number of similar cases have been described to occur without finding specific triggers. For example, Askar et al. reported a case of a young male with a solitary PG arising from a PWS on the cervical area [17]. Also, Sheehan and Lesher described the case of an adult with the co-occurrence of a PG and PWS in the upper dorsum area [11]. Since all of these cases did not show a predisposing factor, an important question arises to understand the underlying mechanism for the growth of a vascular benign tumor in these scenarios. Several efforts have been made to address this issue. In the case reported by Askar et al., they stated that even small traumatic events such as the chronic contact of the patient’s shirt collar led to the development of PG [17]. Similarly, Sheehan and Lesher supported this hypothesis suggesting that this vascular phenomenon is possible when associated with minor traumatic events in the setting of an abnormal vasculature or an underlying place rich in arteriovenous anastomoses [11]. Previous evidence has shown that PG arises more commonly from sites of arteriovenous anastomoses and that PWS and other vascular malformations partly consist of these types of anastomoses, supporting the given conclusions [18]. It is possible that in our case, minor trauma due to clothing friction, possibly not recognized by the patient could be a driver of this clinical presentation.

**Figure 2:**
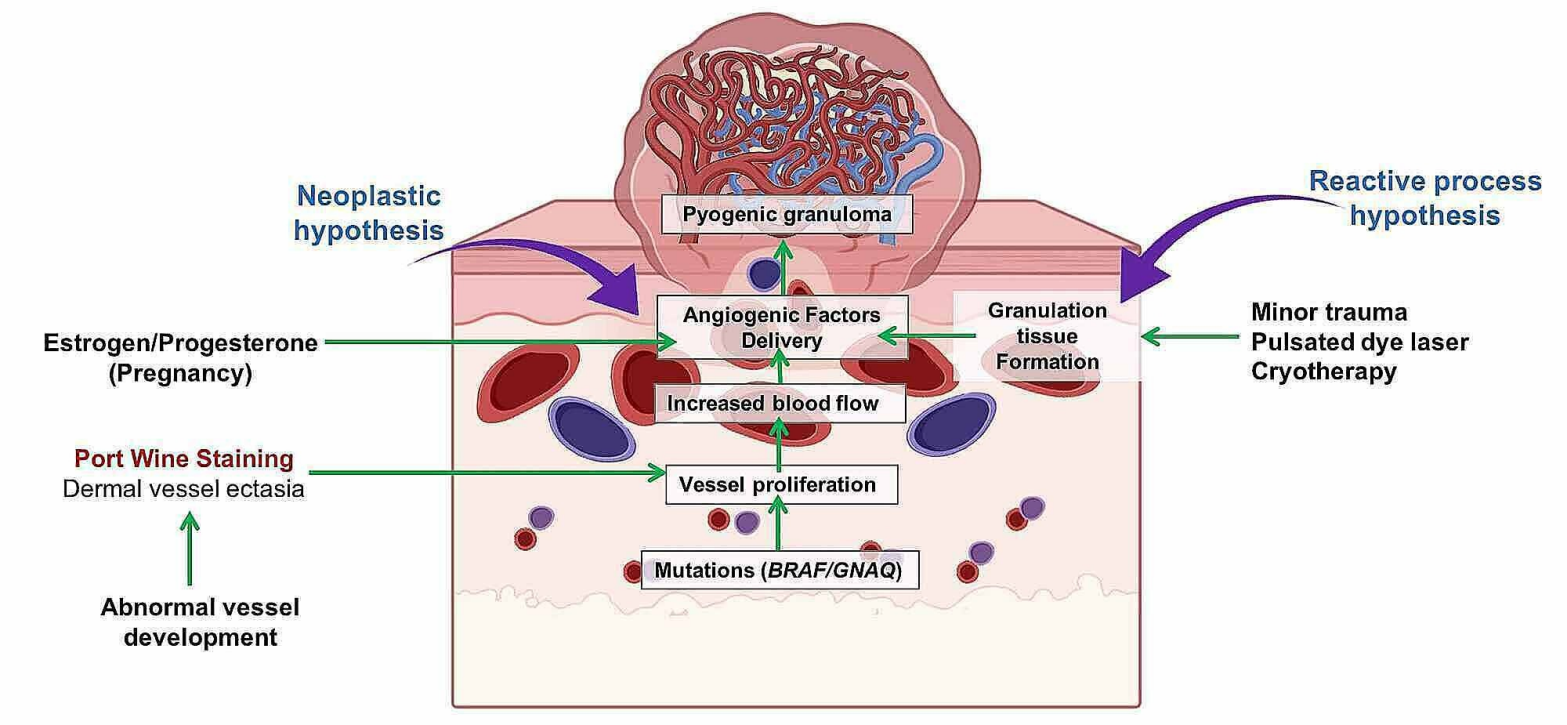
Putative mechanisms involved in the formation of pyogenic granuloma within underlying port wine stain. Original image created by Eder Luna-Ceron by using the BioRender^TM^ drawing platform.

Newer evidence has suggested that PGs emerging from PWS do not result from a mere reactive process. Recently, it has been found that mutations in G protein subunit alpha q (GNAQ) were identified in both PWS and PG, stipulating that PG emerge from the cells of PWS. A specific mutation of v-raf murine sarcoma viral oncogene homolog B1 (BRAF) was found as a major contributing factor for the growth of PG arising from PWS in comparison with spontaneous PG without underlying vascular lesions. This information suggests that the elusive mechanism for the development of PG in the absence of triggers could be explained by a multistep tumor model, in which mutations drive the rapid proliferation of endothelial cells [19]. We believe that minor trauma in the context of a susceptible tissue with mutation hallmarks can contribute to the association of PG and PWS. The possible mechanisms involved in the co-occurrence of PWS and PG are summarized in Figure 2.

Diverse treatments have been suggested as potential therapies for PG including sclerotherapy, laser ablation, beta-blockers, and surgical excision. PG arising from PWS have been reported to have a high recurrence rate and the treatment modality for this lesions should be carefully selected [12]. Since the administration of invasive cutaneous therapies such as laser ablation may induce further trauma and potentially allow recurrence, surgical excision has been favored as the primary treatment in zones without important cosmetic concerns [20].

## Conclusions

As depicted in the previous discussion, PWS are common vascular lesions associated with multiple comorbidities, especially when they appear in the facial region. In this regard, clinicians should assess these entities by making a complete physical examination including a neurological exam. In the cases of PG arising from PWS, it is important to make an oriented interrogatory focusing on finding the presence of the risk factors associated with this clinical picture as pregnancy, trauma, or invasive cutaneous therapies. Furthermore, since the co-occurrence of PG and PWS has been reported to present multiple relapses and since certain types of treatment strategies such as pulsed-dye laser or cryotherapy have been reported to increase recurrence, clinicians should carefully select the treatment modality. Up to date, the treatment of choice has been the surgical excision of the lesion; nevertheless, further research is needed to evaluate new treatment methods to reduce the high rates of reoccurrence events. Although the co-occurrence of PG and PWS has been described as a non-common event, evidence shows that there is a clear relationship between those lesions and that they are not aisled phenomena. Additional research is needed to explore if the etiology of PG that results from PWS responds to a reactive mechanism due to minor trauma or if intrinsic anomalies and mutations leading to the neoplastic formation of PG. In the case presently reported, it is likely that minor trauma, possibly not recognized by the patient, and the existence of intrinsic molecular anomalies associated with PWS might be the drivers for the development of this clinical scenario.
